# Gut Microbiota as an Objective Measurement for Auxiliary Diagnosis of Insomnia Disorder

**DOI:** 10.3389/fmicb.2019.01770

**Published:** 2019-08-13

**Authors:** Bingdong Liu, Weifeng Lin, Shujie Chen, Ting Xiang, Yifan Yang, Yulong Yin, Guohuan Xu, Zhihong Liu, Li Liu, Jiyang Pan, Liwei Xie

**Affiliations:** ^1^Department of Psychiatry, The First Affiliated Hospital of Jinan University, Guangzhou, China; ^2^State Key Laboratory of Applied Microbiology Southern China, Guangdong Provincial Key Laboratory of Microbial Culture Collection and Application, Guangdong Open Laboratory of Applied Microbiology, Guangdong Institute of Microbiology, Guangdong Academy of Sciences, Guangzhou, China; ^3^Department of Infectious Diseases, Nanfang Hospital, Southern Medical University, Guangzhou, China; ^4^Zhujiang Hospital, Southern Medical University, Guangzhou, China

**Keywords:** insomnia, random forest, artificial neural network, redundancy analysis, cross validation

## Abstract

Insomnia is a type of sleep disorder which is associated with various diseases’ development and progression, such as obesity, type II diabetes and cardiovascular diseases. Recent investigation of the gut-brain axis enhances our understanding of the role of the gut microbiota in brain-related diseases. However, whether the gut microbiota is associated with insomnia remains unknown. In the present investigation, leveraging the 16S rDNA amplicon sequencing of V3-V4 region and the novel bioinformatic analysis, it was demonstrated that between insomnia and healthy populations, the composition, diversity and metabolic function of the gut microbiota are significantly changed. Other than these, redundancy analysis, co-occurrence analysis and PICRUSt underpin the gut taxa composition, signaling pathways, and metabolic functions perturbed by insomnia disorder. Moreover, random forest together with cross-validation identified two signature bacteria, which could be used to distinguish the insomnia patients from the healthy population. Furthermore, based on the relative abundance and clinical sleep parameter, we constructed a prediction model utilizing artificial neural network (ANN) for auxiliary diagnosis of insomnia disorder. Overall, the aforementioned study provides a comprehensive understanding of the link between the gut microbiota and insomnia disorder.

## Introduction

Sleep disorder is associated with various diseases’ development and progression, such as obesity, type II diabetes (Knutson et al., 2007) and cardiovascular diseases (Drager et al., 2017). Insomnia is the most prevalent sleep disorder, including sleep apnea, restless legs syndrome (RLS) and narcolepsy, and affects a large proportion of the population on a situational, recurrent or chronic basis and is also one of the most common complaints in medical practice (Morin et al., 2015). The signature of insomnia is that patients have difficulty falling asleep, or staying awake, despite plenty of opportunity to sleep. Like many other psychiatric disorders, insomnia is a multifactorial disorder, though the detailed pathological aspects of insomnia remain unclear. Thus, a better understanding of the pathophysiology of insomnia may provide additional therapeutic strategies.

The gut microbiome, a key component of the intestinal environment, has been implicated as an essential modulator for human health (Tilg et al., 2016). Microbial homeostasis is critical to the host development and health. Dysbiosis perturbs the host immune system and metabolism balance, which leads to the development of various kinds of diseases (Cho and Blaser, 2012; Nieuwdorp et al., 2014; Thaiss et al., 2016). Microbial dysbiosis may also contribute to the development of neurological disorders and psychiatric disorders, such as autism spectrum disorder, anxiety disorder, depression and Alzheimer’s disease (Neufeld et al., 2014; Pistollato et al., 2016; Chen et al., 2017; Lach et al., 2018). In addition, several studies have provided preliminary evidence for the involvement of the gut microbiota in sleep disorders of murine models and human patients. It was reported that after 4 weeks of sleep fragmentation in experimental mice, gut flora were dominated by Lachnospiraceae and Ruminococcaceae, with a gradually reduced relative abundance of Lactobacillaceae (Poroyko et al., 2016). In another study with partial sleep deprivation in normal-weighted young individuals, the composition of the gut microbiota was subtly affected with an increased ratio of Firmicutes/Bacteroidetes (Benedict et al., 2016). However, either sleep fragmentation or sleep deprivation refers to curtailed sleep length due to an externally imposed restriction of the opportunity to sleep, while insomnia refers to the inability to fall asleep adequately, either in length or quality. Considering the significant difference in definition between sleep fragmentation/deprivation and insomnia, to date, study investigating the relationship between insomnia and gut flora remains unexplored.

Thus, in the present investigation we combined 16S rDNA amplicon sequencing and innovative bioinformatic analysis to examine the pathological and physiological significance of the gut microbiota between healthy participants and patients suffering insomnia disorder. Leveraging these innovative analyses, such as redundancy analysis, co-occurrence analysis, PICRUSt, random forest and artificial neural networks (ANN), we demonstrated that the gut taxa composition, signaling pathways, and metabolic functions are perturbed in patients with insomnia disorder. Artificial neural networks were also incorporated by utilizing the relative abundance of the gut microbiota to establish a prediction model for an unbiased evaluation of insomnia. This study is the first to combine high-throughput sequencing and bioinformatic analysis, especially machine learning, to systemically understand the biological effect of the gut microbiota on insomnia. Comprehensive analysis indicated that gut microbiota homeostasis is a strong determinant, which is closely associated with insomnia disorder. Overall, the aforementioned study provides a comprehensive understanding of the link between gut microbiota and insomnia disorder. By utilizing the machine learning approach, we identified the signature gut microbiota, which could be utilized as novel and unbiased prediction targets, which in other aspects could provide additional interventions for clinical application.

## Materials and Methods

### Volunteer Enrollment

The experiment was approved by the Ethics Committee of Jinan University (Approval #: GNU-20180306).

The volunteers were recruited from the public and The First Affiliated Hospital of Jinan University in Guangzhou, China. After being informed on the rights and obligation, all participants understood the benefits and risks of the experiment totally and signed an informed consent document. In compliance with strict standards for inclusion and exclusion criteria (Detailed in Supplementary Materials), all participants were assessed by two psychiatrists. In the event of any dispute or difference of judgment, the participant would be excluded. All participants accepted polysomnography treatment at the Sleep Medicine Center of Jinan University. Finally, twenty qualified volunteers were enrolled and separated into two groups (Insomnia group and Normal Control group). Their fecal samples were collected by sterilized instruments in the morning upon polysomnography treatment, and then stored in a freezer at −80°C for 16S rDNA sequencing.

### 16S rDNA Amplicon Sequencing

Bacterial DNA from patients’ feces was extracted by utilizing the ZR Fecal DNA Kit (Zymo Research, United States). A multiplexed amplicon library covering the V3-V4 region of 16S rDNA gene was PCR-amplified with the optimized primer sets for the Illumina HiSeq 2500 sequencing instrument. A total of 1,534,966 high-quality reads were obtained, with an average of 76,748 reads (range 66,570–84,443) per sample. All chimera sequences were removed by VSEARCH (Quince et al., 2016). Chimera-free sequences were processed using a standard QIIME 1.91 pipeline (Caporaso et al., 2010) and clustered into operational taxonomic units (OTUs) at a 97% similarity threshold using an “Open-Reference” approach. Taxonomy was assigned using the RDP classifier against the Greengenes database (May 2013 release) (McDonald et al., 2012). The raw Illumina pair-end read data for all samples have been deposited in the Short Read Archive under the Bioproject: PRJNA527914.

### Bioinformatics Analysis

Alpha rarefaction was analyzed by the Faith’s phylogenetic diversity (Faith, 1992), Chao1 (Chao, 1984), Shannon and Simpson index (Chao and Shen, 2003). β-diversity was estimated by computing weighted and unweighted UniFrac distance. Principal Coordinates Analysis (PCoA), Redundancy Analysis (RDA) and heatmap of correlation were plotted by “ggplot2,” “vegan,” and “corrplot” packages of R (version 3.5.1). Manhattan Plot was plotted by “edgeR,” “dplyr” and “ggplot2” to present the differential relative abundance between groups. These results were tested by Monte Carlo permutation and Student’s *t*-test. Organism-level microbiome phenotype prediction was obtained by BugBase software (Riaz et al., 2017). To decipher the difference of microbiota structure between groups, LEfSe (linear discriminant analysis effect size) was performed and the cladogram was graphed with default parameter (*p* < 0.05 and LDA score > 2.0) (Fisher, 1936). To probe the microbial metabolism and predict metagenome functional content from the marker gene, PICRUSt was utilized to explore differences of the KEGG pathway between groups (Langille et al., 2013). To decipher the gut microbiota ecology, co-occurrence analysis was performed with the “igraph” package (Nordhausen, 2015) of R with data filtered at species level considering only those relative abundance present in at least 70% of the samples in each group. The edges were estimated by Spearman confident index (abs(*r*) > 0.6, *p* < 0.05). Communities inside two networks were determined by the fast-greedy modularity optimization algorithm (Clauset et al., 2004), which was one of the approaches to determine the dense subgraph in Graph Theory. The circle bar was plotted according to the eigenvector centrality scores (ECS) to estimate the importance and betweenness of each node (Ruhnau, 2000). To identify the key signature microbiota, five-fold cross validation together with Random Forest analysis were performed to compute importance scores (mean decrease accuracy, MDA) to estimate the importance of variables by utilizing the “randomForest” v.4.6-14 package (Breiman, 2001) in R. At species level, in order to establish a prediction model to predict the sleep-related physiological parameter, the ANN was performed on python 3.6.1 with the pyTorch, sklearn, pandas, and numpy packages. The optimized parameters, including learning rate, activation function, layers, number of neurons and dropout, were selected by grid search and cross-validation.

## Results

### Insomnia Disorder Leads to Significant Structural and Functional Changes of Gut Microbiota

**FIGURE 1:**
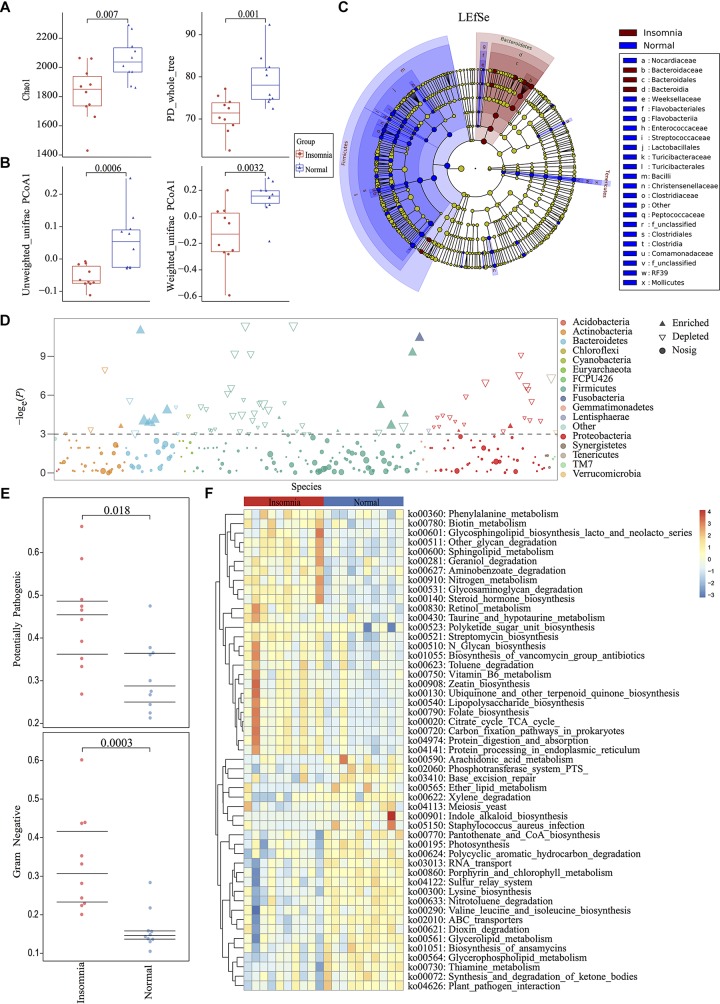
Insomnia disorder leads to significant structural and functional changes of the gut microbiota. **(A)** α-diversity on Chao1 and PD_whole_tree index between insomnia and normal group. **(B)** β-diversity on unweighted and weighted UniFrac PCoA1 between groups. **(C,D)** Manhattan plot and Linear Discriminant Analysis (LDA) Effect Size (LEfSe) plot with threshold for LDA score 2.0 showed significant structural difference and identified 87 biomarkers between the two groups. **(E)** BugBase algorithm predicted the microbiome phenotypes of the insomnia group differed from the normal group on gram-negative and potential pathogenicity significantly. **(F)** To predict the metagenome function, heatmap of PICRUSt analysis showed significant KEGG pathway between groups.

Among the twenty qualified enrolled volunteers, basic personal information including height, weight and BMI presented no significant difference between groups except for age (insomnia: 33.00 ± 6.90; normal: 26.10 ± 1.85) (Supplementary Figure S1). Considering previous research demonstrated the gut microbiota differed little in adults based on more than 1,000 very healthy Chinese individuals (Bian et al., 2017), only 7 mean-years of difference between groups could be tolerated. All the volunteers were accepted according to inclusion and exclusion criteria (detailed in Supplementary Materials). All fecal samples from participants were collected for high-throughput sequencing. 16S rDNA V3-V4 region amplicon sequencing generated 1,534,966 high-quality reads, with an average of 76,748 reads (range 66,570–84,443) per sample. All raw data were filtered by VSEARCH and processed using a standard QIIME 1.91 pipeline against the Greengenes database (May 2013 release). Rarefaction measurement of Shannon and Simpson index, Goods_Coverage, and species accumulation curve (SAC) indicated that sequencing depth was enough to capture all bacterial species and sufficient for downstream analysis (Supplementary Figure S2). Rarefaction analysis of chao1 (*p* = 0.007) and PD whole tree (*p* = 0.001) index showed significant difference between the healthy and insomnia groups, suggesting that insomnia disorder may result in alteration of gut microbiota diversity (Figure 1A). Furthermore, β-diversity calculated with the Unweighted UniFrac (*p* = 0.0006) and Weighted UniFrac (*p* = 0.0032) algorithms indicated that the insomnia and normal groups had significant structural difference by the first dimension of space distance (Figure 1B). To confirm the composition of difference between two groups, a Manhattan plot was used to represent the fold change of insomnia/normal group and revealed a significant difference, especially the Firmicutes and Bacteroidetes phylum, which was confirmed by Linear Discriminant Analysis Effect Size (LEfSe) analysis and identified 87 biomarkers (Figures 1C,D). Meanwhile, BugBase algorithm-based prediction suggested that the insomnia group preferentially enriched with the gram-negative and potential pathogenic taxa compared with the normal group (Figure 1E and Supplementary Figures S3A–F). Other than the composition and diversity of gut microbiota, PICRUSt algorithm was performed to assess the functional difference by plotting the differential pathways against KEGG database. We identified pathways such as steroid hormone biosynthesis (ko00360), Retinol metabolism (ko00830), Vitamin B6 metabolism (ko00750), Folate biosynthesis (ko00790), Citrate cycle TCA cycle (ko00020) that were predicted to be enriched in the insomnia group (Kruskal test *p* < 0.05), while Arachidonic acid metabolism (ko00590), Pantothenate and CoA biosynthesis (ko00770), Lysine biosynthesis (ko00300), and Glycerolipid metabolism (ko00561) associated pathways were downregulated (Kruskal test *p* < 0.05) (Figure 1F).

### Insomnia Disorder Disturbs the Gut Flora Interaction

**FIGURE 2:**
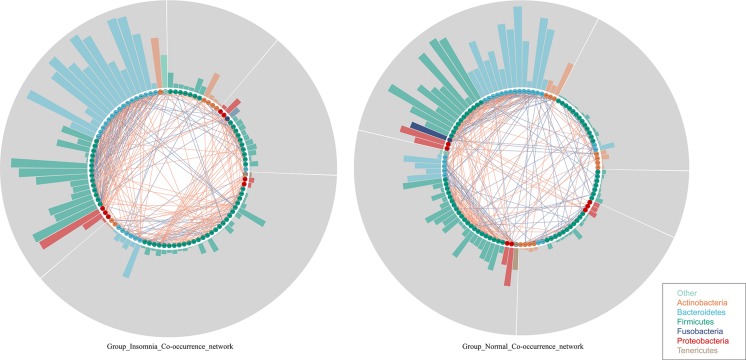
Insomnia disorder disturbs the gut flora interaction. Co-occurrence network described interaction among bacterial taxa. Each node annotated on phylum level presents bacterial species in each group. Each edge colored by the blue and red line indicates the negative and positive interaction between each node, respectively. Each segment (gray color) represents sub-community in networks. Each bar shows the betweenness of each node in each network.

Whether insomnia disorder is associated with the gut microbiota community network and the network complexity, the graph theory algorithm and Co-occurrence analysis were performed to estimate the gut microbiota ecology between groups. The radar plot computed by the graph theory analysis including the transitivity, graph density, degree centralization, number of vertices and number of edges showed that insomnia disorder did not significantly change the systemic complexity of gut bacteria, indicating that the gut microbiota in insomnia patients had already developed a mature network. With this, based on species data whose relative abundance presented at least 70% of the samples in each group, Co-occurrence analysis was used to further explore the gut microbiota interaction and sub-groups in both the normal and insomnia groups (Supplementary Figure S4 and Supplementary Table S1). The gut flora interaction network was significantly altered for patients under insomnia disorder compared with that of the normal group. Furthermore, the gut microbiota was sub-divided into five and four sub-groups for the normal and insomnia groups, respectively (Figure 2).

### Gut Microbiota Alteration Strongly Associated With Insomnia Disorder

**FIGURE 3:**
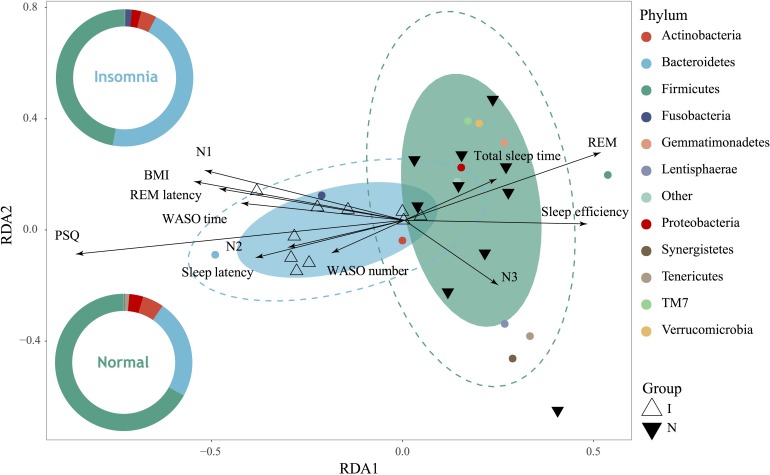
Gut microbiota dysbiosis strongly associated with insomnia disorder. RDA showed high correlation between gut microbiota on phylum level and clinical sleep parameter including polysomnography (PSG) and psychological scale including Pittsburgh sleep quality index (PSQ), body mass index (BMI), total sleep time, sleep efficiency, sleep latency, the number and time of waking after sleep onset (WASO number, WASO time), rapid eye movement (REM), rapid eye movement latency (REM latency), and non-rapid eye movements stages (N1, N2, N3).

As demonstrated above, significant structure, composition and function of the gut microbiota as well as the bacterial interaction network were significantly changed between the normal and insomnia groups. To further prove whether the insomnia-associated clinical sleep parameter directly contributes to the alteration of the gut microbiota, we performed the redundancy analysis (RDA) to link the insomnia parameter with the relative abundance of gut microbiota at phylum level (Figure 3). These clinical sleep parameters from polysomnography (PSG) and the psychological scale include the Pittsburgh Sleep Quality index (PSQ), Hamilton Anxiety Scale (HAMA), Hamilton Depression Scale (HAMD), Epworth Sleepiness Scale (ESS) and Insomnia Severity Index (ISI). Here, we demonstrated that 67.13% of the variance could be interpreted by twelve environmental factors (in other words: clinical sleep parameter), which means that insomnia disorder could significantly alter the population of the gut microbiota at phylum level and samples from two groups were obviously separated. In particular, according to the Monte Carlo permutation test, some clinical sleep parameters, e.g., Pittsburgh sleep quality index (PSQ, *r^2^* = 0.6074, *p* = 0.002) and rapid eye movement sleep (REM, *r^2^* = 0.2663, *p* = 0.045), play a pivotal role in clustering the distribution of flora between groups. Meanwhile, ANOSIM based on the Bray Curtis distance also confirmed the observation from RDA analysis that the difference between groups was more significant than that within groups (statistic *R*: 0.1944, *p* = 0.015) (Supplementary Figure S5). Both RDA and ANOISM analysis clearly suggested that clinical sleep parameters associated with insomnia disorder directly contribute to the separation and clustering of the gut microbiota between groups.

### Identification of the Signature Gut Microbiota Associated With Insomnia Disorder by Random Forest

**FIGURE 4:**
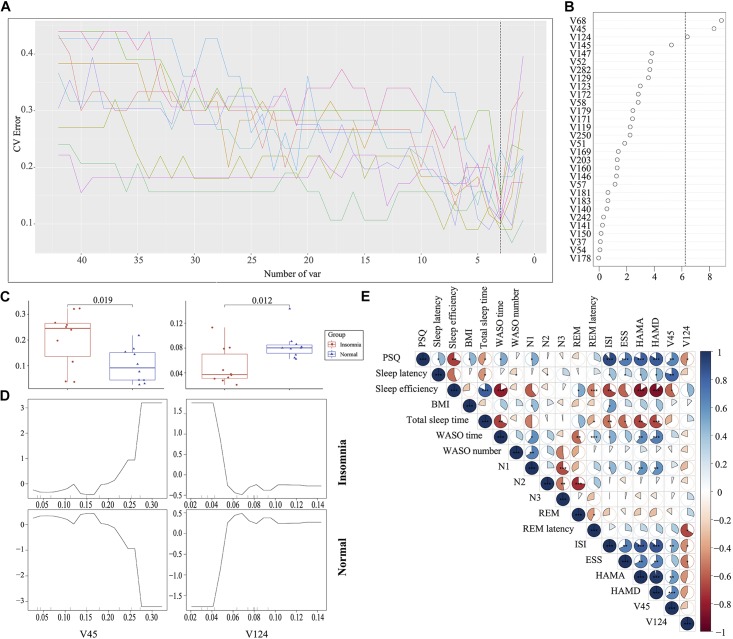
Identification of the signature gut microbiota associated with insomnia disorder by random forest. **(A,B)** To identify the signature biomarkers, five-fold cross-validation together with random forest generating ∼2 million decision trees was performed in the discovery mode. **(C)** Considering the box plot of their relative abundances, two species taxa were selected as the key biomarker. **(D)** The partial dependence of classification in random forest were plotted. **(E)** The correlation analysis between signature taxa and clinical sleep parameter was plotted.

The traditional approaches such as LEfSe by comparing the difference of relative abundance of gut flora between groups resulted in the identification of 87 biomarkers. It is difficult to utilize these markers to establish a prediction model for disease diagnosis. To improve the biomarker identification, we incorporated a robust statistical analysis and applied five-fold cross-validation together with random forest to generate ∼2 million decision trees (Supplementary Figure S6), leading to identification of three optimal species biomarkers with consideration of lowest error rate plus standard deviation. With further analysis to identify V68 (g__Prevotella) as an outlier, we thus selected V45 (g__Bacteroides) and V124 (o__Clostridiales) as the most important biomarkers to distinguish the insomnia patients from healthy individuals with a ROC curve at AUC = 0.87 (Figures 4A–D, Supplementary Figure S7, and Supplementary Table S1). Moreover, V45 was highly correlated with HAMD (*r* = 0.70, *p* < 0.001), HAMA (*r* = 0.62, *p* < 0.01), ISI (*r* = 0.62, *p* < 0.01), sleep efficiency (*r* = −0.56, *p* < 0.05), PSQ (*r* = 0.63, *p* < 0.01) and sleep latency (*r* = 0.66, *p* < 0.01) while V124 correlated with ESS (*r* = −0.45, *p* < 0.05), ISI (*r* = −0.48, *p* < 0.05), REM latency (*r* = −0.49, *p* < 0.05), and PSQ (*r* = −0.51, *p* < 0.05) (Figure 4E and Supplementary Figure S8). Even in the Co-occurrence plot, these two key microbiotas both occupied hub-like positions with high betweenness (Insomnia: V45 0.702499227 V124 0.034447479; Normal: V45 0.467046396 V124 0.044448542) (Supplementary Figure S9). All above results strongly demonstrated that the key microbiota we identified via the robust statistical approaches led to the development of an optimal and robust prediction model for insomnia diagnosis.

### Relative Abundance of Gut Microbiota-Based Prediction on the Clinical Sleep Parameter

**FIGURE 5:**
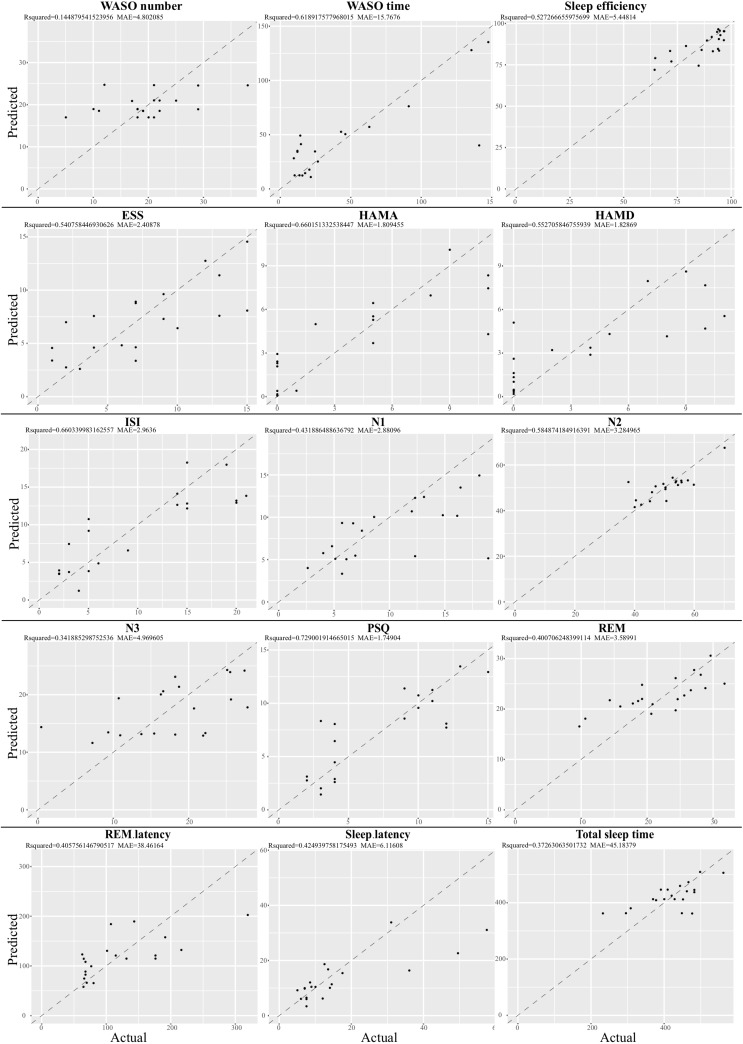
Relative abundance of gut microbiota-based prediction on the clinical sleep parameter by artificial neuron network (ANN). Based on species data, ANN was established to predict clinical sleep parameter with optimized parameter including layers, neuron number, learning rate, dropout rate, and activation function in 5-fold cross validation. WASO, Wake after sleep onset; ESS, Epworth Sleepiness Scale; HAMA, Hamilton Anxiety Rating Scale; HAMD, Hamilton Depression Rating Scale; ISI, Insomnia Severity Index; PSQ, Pittsburgh Sleep Quality Index; REM, Rapid eye movement; N1, Non-rapid eye movement stage 1; N2, Non-rapid eye movement stage 2; N3, Non-rapid eye movement stage 3.

Given that the gut microbiota tightly was correlated with the clinical sleep parameter, we sought to establish a mathematical model to utilize the relative abundance of the gut microbiota to predict the sleep-related physiological parameter. Here, we utilized a well-established regression model, LASSO regression to link the relative abundance of gut microbiota and clinical sleep parameter resulted in a poor correlation (Supplementary Figure S10). To overcome the shortcoming of the traditional machine learning model, we integrated an even more powerful deep learning model, called an ANN, which is considered to be able to imitate biological neural networks. By integrating the clinical sleep parameter into the ANN model, this model could result in a high coefficient of determination respective for WASO number: *r^2^* = 0.14, MAE = 4.80; WASO time: *r^2^* = 0.6, MAE = 15.77; Sleep efficiency: *r^2^* = 0.52, MAE = 5.45; ESS: *r^2^* = 0.54, MAE = 2.41; HAMA: *r^2^* = 0.66, MAE = 1.81; HAMD: *r^2^* = 0.55, MAE = 1.83; ISI: *r^2^* = 0.66, MAE = 2.96; N1: *r^2^* = 0.43, MAE = 2.88; N2 *r^2^* = 0.58, MAE = 3.28; N3: *r^2^* = 0.34, MAE = 4.97; PSQ: *r^2^* = 0.73, MAE = 1.75; REM *r^2^* = 0.40, MAE = 3.59; REM latency: *r^2^* = 0.41, MAE = 38.5; Sleep latency: *r^2^* = 0.42, MAE = 6.12; Total sleep time: *r^2^* = 0.37, MAE = 45.18 (Figure 5).

## Discussion

Insomnia disorder as a common clinical symptom is a critical part of sleep disorder (Panossian and Avidan, 2009). It is often accompanied by excessive arousal and sleep debt, which always lead to adverse impacts such as mental or physical fatigue. From the statistics of more than 50 epidemiological studies, the prevalence of insomnia symptoms was estimated at 10∼48% (Ohayon, 2002). Insomnia disorder is functionally linked to cardiovascular and nervous system diseases (Javaheri and Redline, 2017; Tobaldini et al., 2017). The classic hypothesis is Spielman’s 3P Model including predisposing, precipitating and perpetuating factors (Spielman et al., 1987). Recently, it has been reported that the hypothalamic–pituitary–adrenal axis (HPA) may contribute to the incidence of insomnia (Levenson et al., 2015). Moreover, in 2017 a genome-wide association study (GWAS) identified risk genomic loci and genes that are associated with the incidence of insomnia, and suggested that insomnia is highly polygenic (Hammerschlag et al., 2017). However, none of these studies provided a mechanistic interpretation of the causes or even objective approaches for insomnia diagnosis. Here, our study is first to comprehensively compare the gut microbiota between insomnia patients and healthy individuals. With these data, we established a robust statistical prediction model to utilize the relative abundance of the gut microbiota to distinguish insomnia patients from the normal population and to estimate levels of sleep quality through the novel bioinformatics technology and machine learning algorithm.

The unhealthy shift of gut microbiota, also called dysbiosis, is associated with various metabolic diseases such as obesity, type II diabetes, hypertension and cardiovascular diseases (Clarke et al., 2014; Kristensen et al., 2016; Adnan et al., 2017; Sun et al., 2018). In this study, we demonstrated that α- and β-diversity of the gut microbiota in insomnia patients is significantly altered. Meanwhile, by comparing the difference of the relative abundance between insomnia and healthy individuals, we identified that Bacteroidetes are the dominant taxa in the insomnia group, while Firmicutes and Proteobacteria were enriched in the normal group, resulting in a decreased ratio of Firmicutes/Bacteroidetes. Our results are different from observations made in previous studies in individuals with sleep deprivation or restriction. In their studies, the F/B ratio shows either no change or is increased after partial sleep deprivation (Benedict et al., 2016; Bushman et al., 2017). This discrepancy with respect to the change over the F/B ratio may be due to the difference regarding the clinical definition between sleep restriction/deprivation and insomnia. Sleep deprivation or restriction is not considered to be a specific disease, but rather a result of a wide range of interruption from external environmental factors. It is worth mentioning that although subjects in previous study followed strict experimental protocol, they not only had *ad libitum* access to food/drink throughout the experiment, but also were allowed to read, play video or board games, watch television, and interact with laboratory staff to help remain awake (Bushman et al., 2017). These environmental factors may contribute to variation in the gut microbiota, which leads to difficulty in interpreting the results. Compared to those with sleep deprivation, patients with insomnia who do not have externally imposed restrictions on the opportunity to sleep still have trouble falling asleep, staying asleep, or waking too early, resulting in daytime impairment (Brown, 2005). Our study demonstrated that although insomnia and sleep deprivation may result in similar reductions on sleep length in most cases, they may lead to different consequences regarding the dysbiosis of the gut microbiota. In addition, this ratio change is also reported in different life stages and pathological circumstances. A study looks into the ratio of F/B between adults and elders, suggesting that a higher ratio in the adult gut is observed, while it starts to decrease in individuals undergoing aging (Doré et al., 2009). Alteration of the F/B ratio is also observed in those with metabolic diseases, such as obesity (Turnbaugh et al., 2006; Koliada et al., 2017) and type II diabetes (Vogensen et al., 2010). Furthermore, our BugBase-based phenotypical prediction also demonstrated that the insomnia group was enriched with bacterial taxa to be potentially pathogenic. This may link the insomnia-related sleep disorder population with high potential disease development and progression, as more evidence has proved that chronic sleep disorder is associated with a multitude of health conditions and even systemic metabolic disorder (Van Cauter et al., 2008).

The biological and physiological function of gut microbiota could be defined from multiple aspects, such as the taxonomic composition and diversity, which are poorly conserved across individuals while the genetic composition and functional capacity are evolutionally conserved across individuals. Thus, to decipher the metabolic switch of gut bacteria, the PICRUSt algorithm was utilized to map the bacterial genetic pathway against the KEGG database. Compared to normal group, a wide range of pathways was altered obviously in our study. It is interesting to note that vitamin B-related pathways were significantly induced in the insomnia group, while the level of vitamins is highly associated with the clinical practice of insomnia (Lichstein et al., 2007). In our insomnia patients, the analysis suggested vitamin B6 catabolism (ko00750) in the gut microbiota is significantly enhanced, resulting in vitamin B6 deficiency for the host. It was reported that vitamin B6 is administered as a common therapeutic practice for insomnia disorder and its deficiency results in fatigue and depression (Baldewicz et al., 1998). Thus, additional vitamin B6 supplementation could ameliorate insomnia symptoms (Baldewicz et al., 1998). Moreover, the folate (also called vitamin B9) biosynthesis-related pathway (ko00790) was also increased in the insomnia group. Previous study of serum nutritional biomarkers and dietary supplementation of folate demonstrated that folate acid has a high correlation with the development of sleep disorder (Sato-Mito et al., 2011; Zonderman et al., 2014). In addition, endogenously synthesized arachidonic acid significantly facilitates the release of GABA in the striatum (Chéramy et al., 1996), while GABA could enhance the catabolism of serotonin into N-acetylserotonin (the precursor of melatonin) in rat (Balemans et al., 1983). It has long been speculated that GABA is associated with the synthesis of melatonin and thus might exert regulatory effects on sleep functions. In our study, our bioinformatic analysis demonstrates that arachidonic acid biosynthesis was lower in the insomnia group, indicating that lower production of arachidonic acid from gut microbiota may be associated with a high incidence of insomnia. However, whether arachidonic acid supplementation may improve insomnia symptoms requires further clinical investigation. These results provide a link that gut microbiota and their metabolites maybe a mediator with respect to the development of insomnia. With this information, novel therapeutic and intervention approaches could be developed for people suffering from insomnia disorder in the future.

In our study, insomnia disorder leads to the alteration of the gut microbiota composition and diversity. However, whether insomnia disorder directly contributes to the dysbiosis of the gut microbiota is still unknown. Here, our RDA analysis and ANOSIM provide strong evidence to support the role of clinical sleep parameters of insomnia individuals in dysbiosis of gut microbiota, especially PSQ (*r*^2^ = 0.6074, *p* = 0.002) and REM (*r*^2^ = 0.2663, *p* = 0.045), based on a Monte Carlo permutation test. Both results strongly pinpointed the importance of insomnia disorder as a key factor in separating gut microbiota from two groups. Upon establishment of the link between gut microbiota and clinical sleep parameter, taking advantage of the differential test and LEfSe algorithm, we identified 87 differential biomarkers from the normal and insomnia groups. Among the biomarkers, to further classify their importance, the machine learning approach is incorporated, such as random forest. This robust statistical method could identify the signature biomarkers with higher prediction accuracy and coefficiency, especially for the gut microbiota-based diseases prediction and diagnosis (Ren et al., 2018; Zhu et al., 2018). Here, our random forest model together with the cross-validation model identified two key bacterial taxa (g__Bacteroides; o__Clostridiales), which are not only tightly associated with clinical data, but also play a pivotal role in network of gut ecology and could be used as two critical biomarkers to identify patients with insomnia.

Classic diagnosis for insomnia disorder relies on either subjective or objective assessment, including the most common clinical sleep parameters such as PSQ, ESS, ISI, HAMD, and HAMA. However, most of these results are often affected by the subjectivity of individuals, especially for some patients with insomnia disorder (Åkerstedt and Gillberg, 1990; Landry et al., 2015). On the other hand, PSG, as the first choice for objective assessment, is the golden standard for insomnia diagnosis worldwide. This is restricted by the cost, equipment and space. Furthermore, the adaptation of the first-night sleep may affect the PSG results because of the temporary change of sleep environment (Tamaki et al., 2016). Thus, a convenient approach is necessary for the diagnosis of insomnia. Given the tight correlation between microbiota and disease incidence, whether there is a method to establish a regression model to predict clinical sleep parameter remains unclear. So, we introduced a LASSO regression model, which is widely used in gut microbiota-based clinical study and has been shown to effectively utilize the relative abundance to predict cancer development and progression, such as irritable bowel diseases and colorectal cancer (Tap et al., 2017; Flemer et al., 2018). Moreover, LASSO could overcome the multicollinearity problem caused by the interaction between microbes (Tibshirani, 1996), while regression models were limited in microbiology study (Alin, 2010). However, in our insomnia case, the LASSO model could not collect enough fitness for the current study (Supplementary Figure S10). To overcome the limitation of LASSO regression, we introduced ANN, which was originally developed to imitate the biological neural networks of the brain (McCulloch and Pitts, 1943). ANN is not only an algorithm, but also a frame for different machine learning algorithms to incorporate and work together to process complex data. As it works in the same way as the human brain, compared to traditional machining learning such as LASSO, ANN brought out stronger and more robust ability to deal with complex data, offered a good prediction model with high fitness, and thus was applied to various areas, especially in quantum chemistry (Balabin and Lomakina, 2009), general game playing (Silver et al., 2016), 3D reconstruction (Choy et al., 2016), and medical diagnosis (Kamruzzaman et al., 2004). In these areas, ANN like LASSO regression could also effectively and practically address the multicollinearity problem. Thus, we incorporated an ANN prediction model to assess sleep quality based on the relative abundance of the gut microbiota. Although based on few samples, this model could still obtain good fitness. With this, we are able to utilize the relative abundance of the gut microbiota to provide an alternative and accurate approach for insomnia diagnosis.

## Conclusion

The model proposed in the current study utilizes the cutting edge bioinformatic algorithm to not only underpin the difference between insomnia and normal health, but also take advantage of ANN to establish the prediction model for insomnia diagnosis and sleep quality evaluation based on the relative abundance of the gut microbiota. Although all methods above are only based on bioinformatics and mathematics, we believe these approaches could validate the results and further prove that even with a small sample size. With this, we could still be able to draw a solid conclusion. Of course, more cases will be collected to provide further evidence in our future work. This will open another gate and a new perspective for the development of novel therapeutic strategies by taking advantage of the information from the gut microbiota.

## Data Availability

The generated datasets for this study can be found on BioProject accession number PRJNA527914.

## Ethics Statement

The experiment was proved by the Ethics Committee of Jinan University and recruited volunteers in public and The First Affiliated Hospital of Jinan University in Guangzhou, China (Approval #: GNU-20180306).

## Author Contributions

LX, LL, and JP designed the experiments. LX and JP collected the grant support. BL, SC, and ZL performed the data analysis. WL, TX, GX, YlY, and YfY collected participants’ feces. BL and LX drafted the manuscript.

## Conflict of Interest Statement

The authors declare that the research was conducted in the absence of any commercial or financial relationships that could be construed as a potential conflict of interest.

## Supplementary Material

FIGURE S1 The general information including BMI **(A)**, Height **(B)**, Weight **(C)**, and Age **(D)** between insomnia and normal group.

FIGURE S2 Rarefaction measurement of Shannon **(A)** and Simpson **(B)** index presented a saturate platform indicated sequencing depth was enough to capture all bacterial species while Good’s coverage index **(C)** and species accumulation curve **(D)** confirmed the sampling was sufficient for the experiment design on OTU taxa.

FIGURE S3 BugBase algorithm was used to predict microbiome phenotypes including Gram positive **(A)** or negative **(B)**, aerobic **(C)** or anaerobic **(D)**, Potential_Pathogenic **(E)**, and Forms_Biofilms **(F)** with Mann-Whitney *U* test.

FIGURE S4 Radar plot on transitivity, number of edges, number of vertices, degree of centralization, and graph density indicated the gut microbiota in each group developed a mature network with almost same complexity.

FIGURE S5 Analysis of similarity (ANOSIM) revealed the difference between groups was more significant than that within groups (statistic *R*: 0.1944, *p* = 0.015).

FIGURE S6 The detailed results of random forest in ten different random seed are presented.

FIGURE S7 Based on two key bacterial taxa (V45, V124), the random forest prediction obtained an accurate rate with the ROC curve at AUC = 0.87.

FIGURE S8 The detailed results of correlation analysis, these key taxa and clinical sleep parameter were mapped.

FIGURE S9 Co-occurrence network in each group plotted with “auto layout” parameter in “igraph” packages shows V45 and V124 occupied hub-like position in each network.

FIGURE S10 LASSO regression model to utilize the relative abundance of bacterial taxa to predict clinical sleep parameter.

## References

[B1] Adnan S. Nelson J. W. Ajami N. J. Venna V. R. Petrosino J. F. Bryan R. M. (2017). Alterations in the gut microbiota can elicit hypertension in rats. *Physiol. Genomics* 49 96–104. 10.1152/physiolgenomics.00081.2016 28011881 PMC5336599

[B2] Åkerstedt T. Gillberg M. (1990). Subjective and objective sleepiness in the active individual. *Int. J. Neurosci.* 52 29–37. 10.3109/00207459008994241 2265922

[B3] Alin A. (2010). Multicollinearity. *Wiley Interdiscip. Rev. Comput. Stat.* 2 370–374. 10.1002/wics.84

[B4] Balabin R. M. Lomakina E. I. (2009). Neural network approach to quantum-chemistry data: accurate prediction of density functional theory energies. *J. Chem. Phys.* 131:074104. 10.1063/1.3206326 19708729

[B5] Baldewicz T. Goodkin K. Feaster D. J. Blaney N. T. Kumar M. Kumar A. (1998). Plasma pyridoxine deficiency is related to increased psychological distress in recently bereaved homosexual men. *Psychosom. Med.* 60 297–308. 10.1097/00006842-199805000-00016 9625217

[B6] Balemans M. G. Mans D. Smith I. Van Benthem J. (1983). The influence of GABA on the synthesis of N-acetylserotonin, melatonin, O-acetyl-5-hydroxytryptophol and O-acetyl-5-methoxytryptophol in the pineal gland of the male Wistar rat. *Reprod. Nutr. Dev.* 23 151–160. 10.1051/rnd:19830114 6844712

[B7] Benedict C. Vogel H. Jonas W. Woting A. Blaut M. Schürmann A. (2016). Gut microbiota and glucometabolic alterations in response to recurrent partial sleep deprivation in normal-weight young individuals. *Mol. Metab.* 5 1175–1186. 10.1016/j.molmet.2016.10.003 27900260 PMC5123208

[B8] Bian G. Gloor G. B. Gong A. Jia C. Zhang W. Hu J. (2017). The gut microbiota of healthy aged chinese is similar to that of the healthy young. *mSphere* 2:e00327-17. 10.1128/mSphere.00327-317 28959739 PMC5615133

[B9] Breiman L. (2001). Random forests. *Mach. Learn.* 45 5–32. 10.1023/A:1010933404324

[B10] Brown W. D. (2005). “Insomnia: prevalence and daytime consequences,” in *Sleep: A Comprehensive Handbook*, ed. Lee-Chiongcpesnm T. L., (Hoboken, NJ: John Wiley & Sons, Inc), 91–98. 10.1002/0471751723.ch12

[B11] Bushman F. D. Bai L. Sehgal A. Goel N. Dinges D. F. Zhang S. L. (2017). Human and rat gut microbiome composition is maintained following sleep restriction. *Proc. Natl. Acad. Sci. U.S.A.* 114 E1564–E1571. 10.1073/pnas.1620673114 28179566 PMC5338418

[B12] Caporaso J. G. Kuczynski J. Stombaugh J. Bittinger K. Bushman F. D. Costello E. K. (2010). QIIME allows analysis of high-throughput community sequencing data. *Nat. Methods* 7 335–336. 10.1038/nmeth.f.303 20383131 PMC3156573

[B13] Chao A. (1984). Nonparametric estimation of the number of classes in a population. *Scand. J. Stat.* 11 265–270.

[B14] Chao A. Shen T. J. (2003). Nonparametric estimation of Shannon’s index of diversity when there are unseen species in sample. *Environ. Ecol. Stat.* 10 429–443. 10.1023/A:1026096204727

[B15] Chen K. Liu Q. Qiu C. Wang J. Luan X. Dan Z. (2017). The histone H3K4 demethylase KDM5 modulate gut microbiota composition to affect social behavior. *Gastroenterology* 152:S821 10.1016/s0016-5085(17)32836-32836

[B16] Chéramy A. Artaud F. Godeheu G. L’Hirondel M. Glowinski J. (1996). Stimulatory effect of arachidonic acid on the release of GABA in matrix-enriched areas from the rat striatum. *Brain Res.* 742 185–194. 10.1016/s0006-8993(96)00963-8 9117394

[B17] Cho I. Blaser M. J. (2012). The human microbiome: at the interface of health and disease. *Nat. Rev. Genet.* 13 260–270. 10.1038/nrg3182 22411464 PMC3418802

[B18] Choy C. B. Xu D. Gwak J. Y. Chen K. Savarese S. (2016). “3D-R2N2: a unified approach for single and multi-view 3D object reconstruction,” in *Lecture Notes in Computer Science (including subseries Lecture Notes in Artificial Intelligence and Lecture Notes in Bioinformatics)*, eds Leibe B. Matas J. Sebe N. Welling M. (Cham: Springer). 10.1007/978-3-319-46484-8_38

[B19] Clarke S. F. Murphy E. F. O’Sullivan O. Lucey A. J. Humphreys M. Hogan A. (2014). Exercise and associated dietary extremes impact on gut microbial diversity. *Gut* 63 1913–1920. 10.1136/gutjnl-2013-306541 25021423

[B20] Clauset A. Newman M. E. J. Moore C. (2004). Finding community structure in very large networks. *Phys. Rev.* 70 1–6. 10.1103/PhysRevE.70.066111 15697438

[B21] Doré J. Guimarăes V. Sokol H. Corthier G. Firmesse O. Mariat D. (2009). The Firmicutes/Bacteroidetes ratio of the human microbiota changes with age. *BMC Microbiol.* 9:123. 10.1186/1471-2180-9-123 19508720 PMC2702274

[B22] Drager L. F. McEvoy R. D. Barbe F. Lorenzi-Filho G. Redline S. (2017). Sleep apnea and cardiovascular disease: lessons from recent trials and need for team science. *Circulation* 136 1840–1850. 10.1161/CIRCULATIONAHA.117.029400 29109195 PMC5689452

[B23] Faith D. P. (1992). Conservation evaluation and phylogenetic diversity. *Biol. Conserv.* 61 1–10. 10.1016/0006-3207(92)91201-91203

[B24] Fisher R. A. (1936). The use of multiple measurements in taxonomic problems. *Ann. Eugen.* 7 179–188.

[B25] Flemer B. Warren R. D. Barrett M. P. Cisek K. Das A. Jeffery I. B. (2018). The oral microbiota in colorectal cancer is distinctive and predictive. *Gut* 67 1454–1463. 10.1136/gutjnl-2017-314814 28988196 PMC6204958

[B26] Hammerschlag A. R. Stringer S. De Leeuw C. A. Sniekers S. Taskesen E. Watanabe K. (2017). Genome-wide association analysis of insomnia complaints identifies risk genes and genetic overlap with psychiatric and metabolic traits. *Nat. Genet.* 49 1584–1592. 10.1038/ng.3888 28604731 PMC5600256

[B27] Javaheri S. Redline S. (2017). Insomnia and risk of cardiovascular disease. *Chest* 152 435–444. 10.1016/j.chest.2017.01.026 28153671 PMC5577359

[B28] Kamruzzaman S. M. Hasan A. R. Siddiquee A. B. Mazumder E. H. (2004). “Medical Diagnosis Using Neural Network,” in *Proceeding of the 3rd International. Conference Electrical & Computer Engineering*, Dhaka.

[B29] Knutson K. L. Spiegel K. Penev P. Van Cauter E. (2007). The metabolic consequences of sleep deprivation. *Sleep Med. Rev.* 11 163–178. 10.1016/j.smrv.2007.01.002 17442599 PMC1991337

[B30] Koliada A. Syzenko G. Moseiko V. Budovska L. Puchkov K. Perederiy V. (2017). Association between body mass index and Firmicutes/Bacteroidetes ratio in an adult Ukrainian population. *BMC Microbiol.* 17:120. 10.1186/s12866-017-1027-1021 28532414 PMC5440985

[B31] Kristensen N. B. Bryrup T. Allin K. H. Nielsen T. Hansen T. H. Pedersen O. (2016). Alterations in fecal microbiota composition by probiotic supplementation in healthy adults: a systematic review of randomized controlled trials. *Genome Med.* 8:52. 10.1186/s13073-016-0300-5 27159972 PMC4862129

[B32] Lach G. Schellekens H. Dinan T. G. Cryan J. F. (2018). Anxiety, depression, and the microbiome: a role for gut peptides. *Neurotherapeutics* 15 36–59. 10.1007/s13311-017-0585-580 29134359 PMC5794698

[B33] Landry G. J. Best J. R. Liu-Ambrose T. (2015). Measuring sleep quality in older adults: a comparison using subjective and objective methods. *Front. Aging Neurosci.* 7:166. 10.3389/fnagi.2015.00166 26441633 PMC4561455

[B34] Langille M. G. I. Zaneveld J. Caporaso J. G. McDonald D. Knights D. Reyes J. A. (2013). Predictive functional profiling of microbial communities using 16S rRNA marker gene sequences. *Nat. Biotechnol.* 31 814–821. 10.1038/nbt.2676 23975157 PMC3819121

[B35] Levenson J. C. Kay D. B. Buysse D. J. (2015). The pathophysiology of insomnia. *Chest* 147 1179–1192. 10.1378/chest.14-1617 25846534 PMC4388122

[B36] Lichstein K. L. Payne K. L. Soeffing J. P. Heith Durrence H. Taylor D. J. Riedel B. W. (2007). Vitamins and sleep: an exploratory study. *Sleep Med.* 9 27–32. 10.1016/j.sleep.2006.12.009 17825610 PMC2174691

[B37] McCulloch W. S. Pitts W. (1943). A logical calculus of the ideas immanent in nervous activity. *Bull. Math. Biophys.* 5 115–133. 10.1007/BF02478259 2185863

[B38] McDonald D. Price M. N. Goodrich J. Nawrocki E. P. DeSantis T. Z. Probst A. (2012). An improved greengenes taxonomy with explicit ranks for ecological and evolutionary analyses of bacteria and archaea. *ISME J.* 6 610–618. 10.1038/ismej.2011.139 22134646 PMC3280142

[B39] Morin C. M. Drake C. L. Harvey A. G. Krystal A. D. Manber R. Riemann D. (2015). Insomnia disorder. *Nat. Rev. Dis. Primers* 1:15026. 10.1038/nrdp.2015.26 27189779

[B40] Neufeld K.-A. M. Kang N. Bienenstock J. Foster J. A. (2014). Effects of intestinal microbiota on anxiety-like behavior. *Commun. Integr. Biol.* 4 492–494. 10.4161/cib.15702 PMC3181531 21966581

[B41] Nieuwdorp M. Gilijamse P. W. Pai N. Kaplan L. M. (2014). Role of the microbiome in energy regulation and metabolism. *Gastroenterology* 146 1525–1533. 10.1053/j.gastro.2014.02.008 24560870

[B42] Nordhausen K. (2015). Statistical analysis of network data with R. *Int. Stat. Rev.* 83 171–172. 10.1111/insr.12095_10

[B43] Ohayon M. M. (2002). Epidemiology of insomnia: what we know and what we still need to learn. *Sleep Med. Rev.* 6 97–111. 10.1053/smrv.2002.0186 12531146

[B44] Panossian L. A. Avidan A. Y. (2009). Review of sleep disorders. *Med. Clin. North Am*. 93 407–425. 10.1016/j.mcna.2008.09.001 19272516

[B45] Pistollato F. Cano S. S. Elio I. Vergara M. M. Giampieri F. Battino M. (2016). Role of gut microbiota and nutrients in amyloid formation and pathogenesis of Alzheimer disease. *Nutr. Rev.* 74 624–634. 10.1093/nutrit/nuw023 27634977

[B46] Poroyko V. A. Carreras A. Khalyfa A. Khalyfa A. A. Leone V. Peris E. (2016). Chronic sleep disruption alters gut microbiota, induces systemic and adipose tissue inflammation and insulin resistance in mice. *Sci. Rep.* 6:35405. 10.1038/srep35405 27739530 PMC5064361

[B47] Quince C. Nichols B. Rognes T. Flouri T. Mahé F. (2016). VSEARCH: a versatile open source tool for metagenomics. *PeerJ* 4:e2584. 10.7717/peerj.2584 27781170 PMC5075697

[B48] Ren Z. Li A. Jiang J. Zhou L. Yu Z. Lu H. (2018). Gut microbiome analysis as a tool towards targeted non-invasive biomarkers for early hepatocellular carcinoma. *Gut* 2018:gutjnl–2017–315084. 10.1136/gutjnl-2017-315084 30045880 PMC6580753

[B49] Riaz H. Khan A. R. Khan M. S. Rehman K. A. Alansari S. A. R. Gheyath B. (2017). BugBase predicts organism-level microbiome phenotypes. *Am. J. Cardiol.* 120 774–781. 10.1016/j.amjcard.2017.05.046 28779871

[B50] Ruhnau B. (2000). Eigenvector-centrality - a node-centrality. *Soc. Netw.* 22 357–365. 10.1016/S0378-8733(00)00031-9

[B51] Sato-Mito N. Sasaki S. Murakami K. Okubo H. Takahashi Y. Shibata S. (2011). The midpoint of sleep is associated with dietary intake and dietary behavior among young Japanese women. *Sleep Med*. 12 289–294. 10.1016/j.sleep.2010.09.012 21296614

[B52] Silver D. Huang A. Maddison C. J. Guez A. Sifre L. Van Den Driessche G. (2016). Mastering the game of Go with deep neural networks and tree search. *Nature* 529 484–489. 10.1038/nature16961 26819042

[B53] Spielman A. J. Caruso L. S. Glovinsky P. B. (1987). A behavioral perspective on insomnia treatment. *Psychiatr. Clin. North Am.* 10 541–553. 10.1016/S0193-953X(18)30532-X 3332317

[B54] Sun L. Ma L. Ma Y. Zhang F. Zhao C. Nie Y. (2018). Insights into the role of gut microbiota in obesity: pathogenesis, mechanisms, and therapeutic perspectives. *Protein Cell* 9 397–403. 10.1007/s13238-018-0546-543 29725936 PMC5960470

[B55] Tamaki M. Bang J. W. Watanabe T. Sasaki Y. (2016). Night watch in one brain hemisphere during sleep associated with the first-night effect in humans. *Curr. Biol.* 26 1190–1194. 10.1016/j.cub.2016.02.063 27112296 PMC4864126

[B56] Tap J. Derrien M. Törnblom H. Brazeilles R. Cools-Portier S. Doré J. (2017). Identification of an intestinal microbiota signature associated with severity of irritable bowel syndrome. *Gastroenterology* 152 111–123.e8. 10.1053/j.gastro.2016.09.049 27725146

[B57] Thaiss C. A. Zmora N. Levy M. Elinav E. (2016). The microbiome and innate immunity. *Nature* 535 65–74. 10.1038/nature18847 27383981

[B58] Tibshirani R. (1996). Regression shrinkage and selection via the lasso. *J. R. Stat. Soc. Ser. B* 58 267–288.

[B59] Tilg H. Cani P. D. Mayer E. A. (2016). Gut microbiome and liver diseases. *Gut* 65 2035–2044. 10.1136/gutjnl-2016-312729 27802157

[B60] Tobaldini E. Costantino G. Solbiati M. Cogliati C. Kara T. Nobili L. (2017). Sleep, sleep deprivation, autonomic nervous system and cardiovascular diseases. *Neurosci. Biobehav. Rev.* 74(Pt B), 321–329. 10.1016/j.neubiorev.2016.07.004 27397854

[B61] Turnbaugh P. J. Ley R. E. Mahowald M. A. Magrini V. Mardis E. R. Gordon J. I. (2006). An obesity-associated gut microbiome with increased capacity for energy harvest. *Nature* 444 1027–1031. 10.1038/nature05414 17183312

[B62] Van Cauter E. Spiegel K. Tasali E. Leproult R. (2008). Metabolic consequences of sleep and sleep loss. *Sleep Med.* 9 (Suppl. 1), S23–S28. 10.1016/S1389-9457(08)70013-3 18929315 PMC4444051

[B63] Vogensen F. K. van den Berg F. W. J. Al-Soud W. A. Jakobsen M. Larsen N. Pedersen B. K. (2010). Gut microbiota in human adults with type 2 diabetes differs from non-diabetic adults. *PLoS One* 5:e9085. 10.1371/journal.pone.0009085 20140211 PMC2816710

[B64] Zhu J. Liao M. Yao Z. Liang W. Li Q. Liu J. (2018). Breast cancer in postmenopausal women is associated with an altered gut metagenome. *Microbiome* 6:136. 10.1186/s40168-018-0515-513 30081953 PMC6080540

[B65] Zonderman A. B. Canas J. A. Gamaldo A. A. Beydoun H. A. McNeely J. M. Beydoun M. A. (2014). Serum nutritional biomarkers and their associations with sleep among US adults in recent national surveys. *PLoS One* 9:e103490. 10.1371/journal.pone.0103490 25137304 PMC4138077

