# Corrigendum: Gut Microbiota as an Objective Measurement for Auxiliary Diagnosis of Insomnia Disorder

**DOI:** 10.3389/fmicb.2020.00510

**Published:** 2020-04-02

**Authors:** Bingdong Liu, Weifeng Lin, Shujie Chen, Ting Xiang, Yifan Yang, Yulong Yin, Guohuan Xu, Zhihong Liu, Li Liu, Jiyang Pan, Liwei Xie

**Affiliations:** ^1^Department of Psychiatry, The First Affiliated Hospital of Jinan University, Guangzhou, China; ^2^State Key Laboratory of Applied Microbiology Southern China, Guangdong Provincial Key Laboratory of Microbial Culture Collection and Application, Guangdong Open Laboratory of Applied Microbiology, Guangdong Institute of Microbiology, Guangdong Academy of Sciences, Guangzhou, China; ^3^Department of Infectious Diseases, Nanfang Hospital, Southern Medical University, Guangzhou, China; ^4^Zhujiang Hospital, Southern Medical University, Guangzhou, China

**Keywords:** insomnia, random forest, artificial neural network, redundancy analysis, cross validation

In the original article, there was an error in the article title. There was typo in our accepted research article. The title of the article was “Gut Microbiota as a Subjective Measurement for Auxiliary Diagnosis of Insomnia Disorder.”

The word “subjective” should be “objective.” Thus, the correct title is “Gut Microbiota as an Objective Measurement for Auxiliary Diagnosis of Insomnia Disorder.”

The authors apologize for this error and state that this does not change the scientific conclusions of the article in any way. The original article has been updated.

