# Supplementary figures and images for: Gut Microbiota as an Objective Measurement for Auxiliary Diagnosis of Insomnia Disorder
Source: Front Microbiol. 2019 Aug 13;10:1770. doi: 10.3389/fmicb.2019.01770 (PMC6701205; doi:10.3389/fmicb.2019.01770)

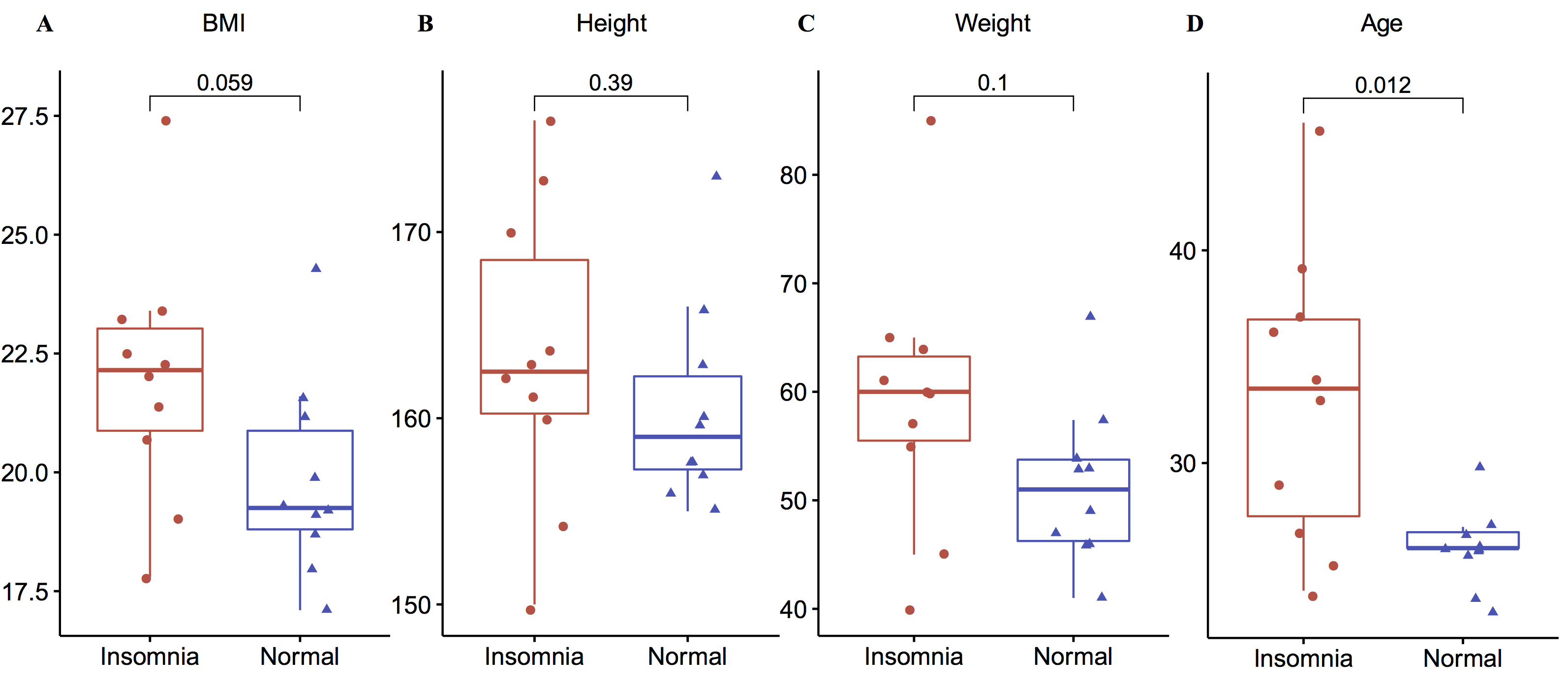

Supplement: FIGURE S1 — The general information including BMI (A), Height (B), Weight (C), and Age (D) between insomnia and normal group. [file Image_1.JPEG]

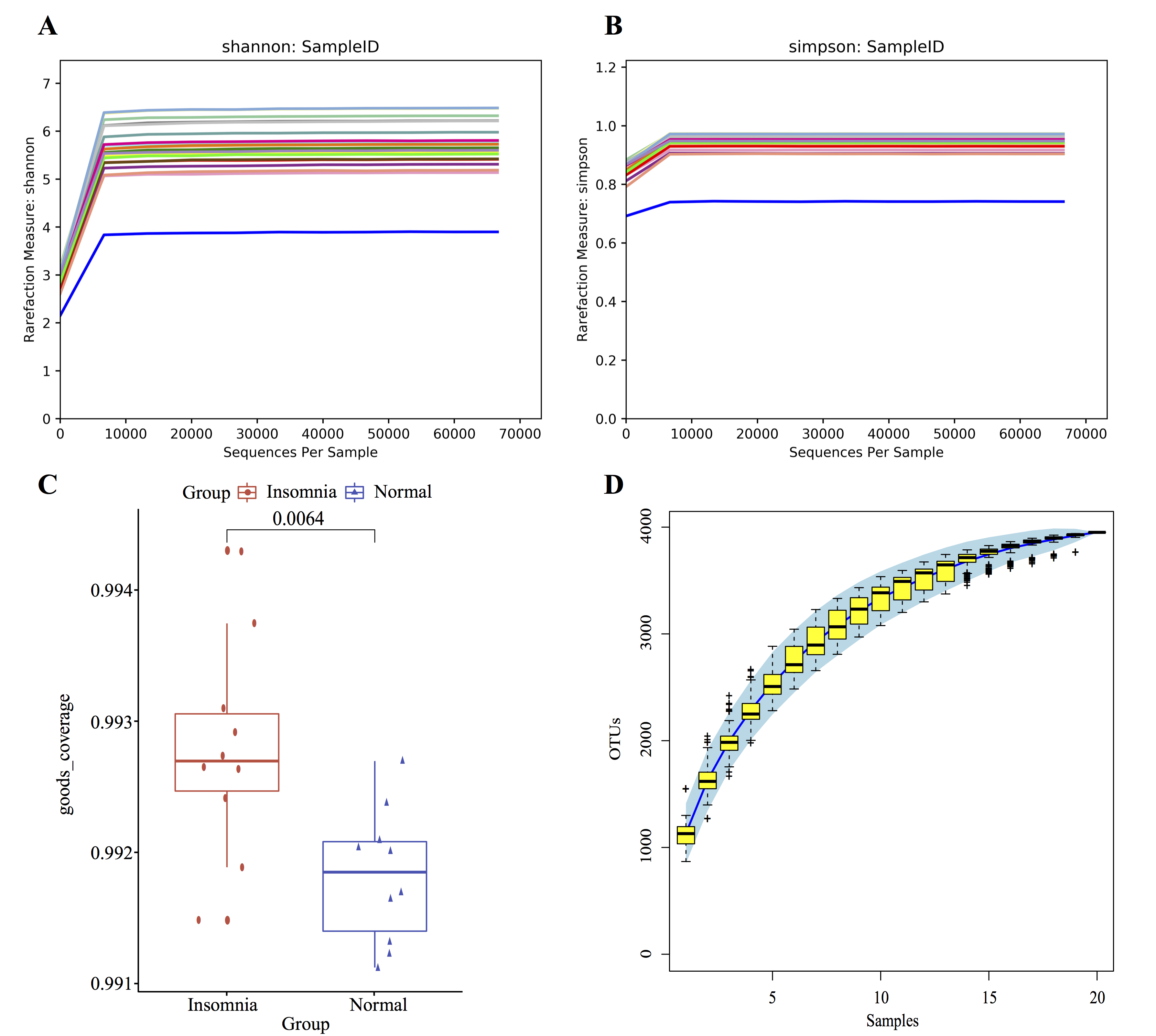

Supplement: FIGURE S2 — Rarefaction measurement of Shannon (A) and Simpson (B) index presented a saturate platform indicated sequencing depth was enough to capture all bacterial species while Good’s coverage index (C) and species accumulation curve (D) confirmed the sampling was sufficient for the experiment design on OTU taxa. [file Image_2.JPEG]

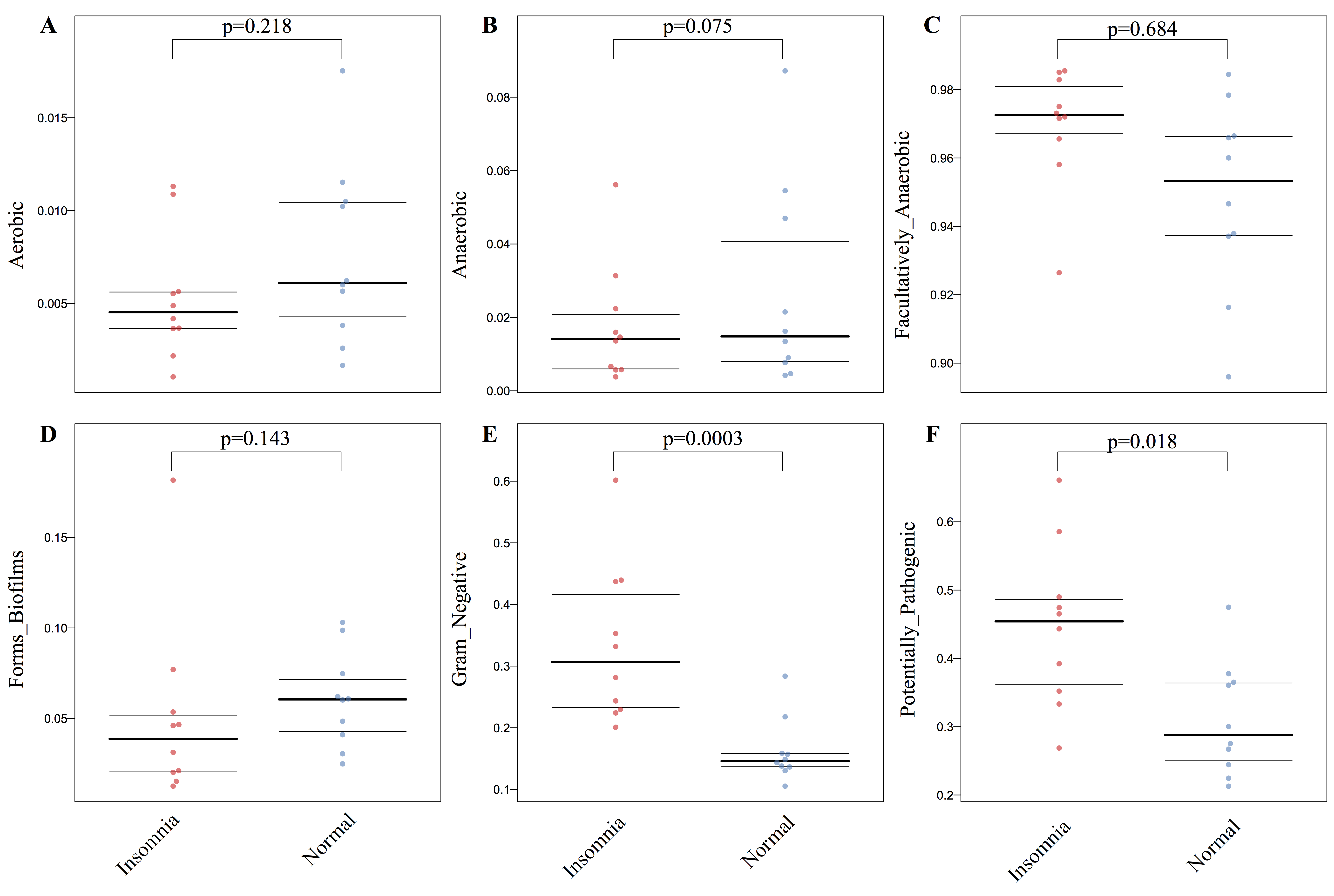

Supplement: FIGURE S3 — BugBase algorithm was used to predict microbiome phenotypes including Gram positive (A) or negative (B), aerobic (C) or anaerobic (D), Potential_Pathogenic (E), and Forms_Biofilms (F) with Mann-Whitney U test. [file Image_3.JPEG]

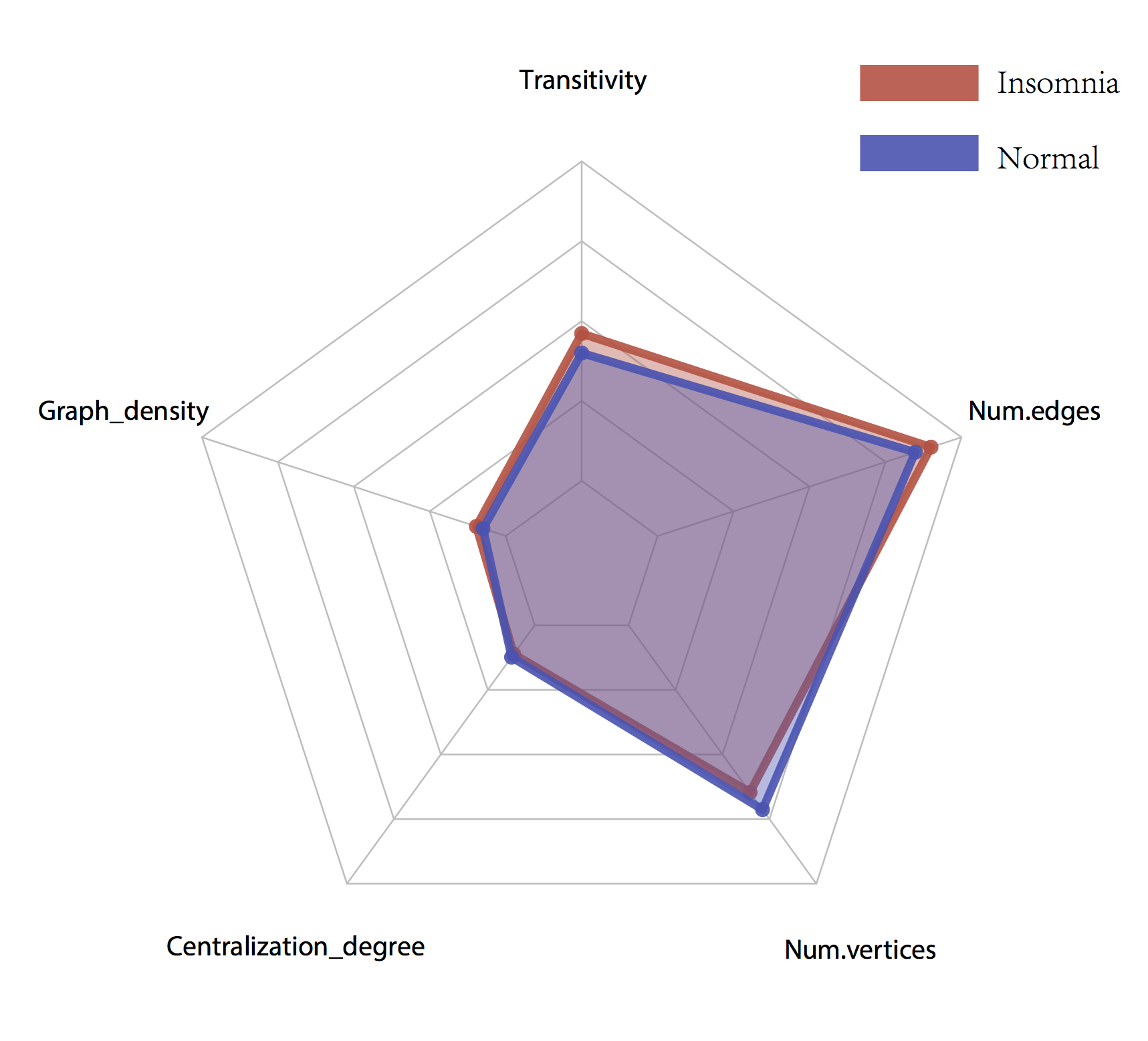

Supplement: FIGURE S4 — Radar plot on transitivity, number of edges, number of vertices, degree of centralization, and graph density indicated the gut microbiota in each group developed a mature network with almost same complexity. [file Image_4.JPEG]

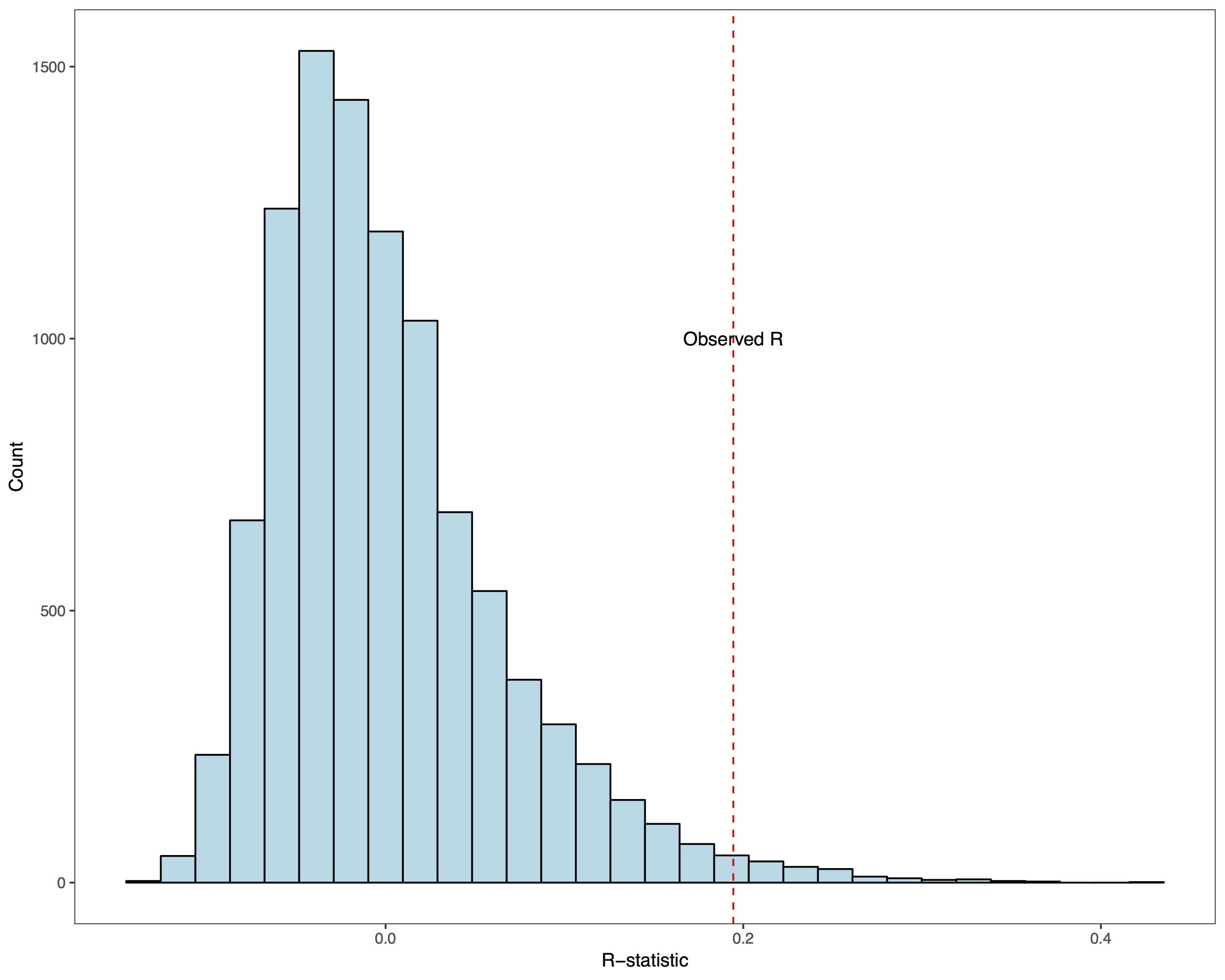

Supplement: FIGURE S5 — Analysis of similarity (ANOSIM) revealed the difference between groups was more significant than that within groups (statistic R: 0.1944, p = 0.015). [file Image_5.JPEG]

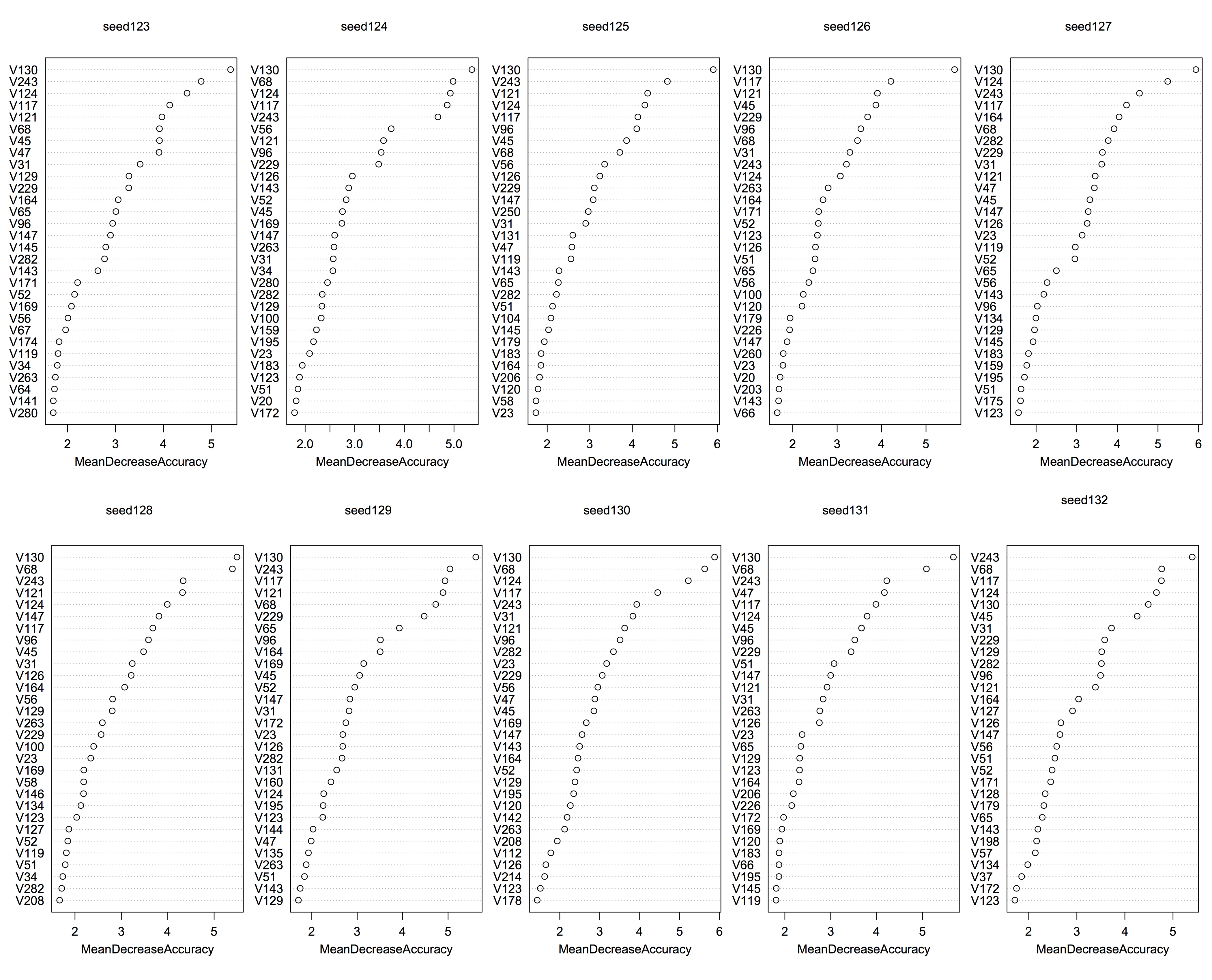

Supplement: FIGURE S6 — The detailed results of random forest in ten different random seed are presented. [file Image_6.JPEG]

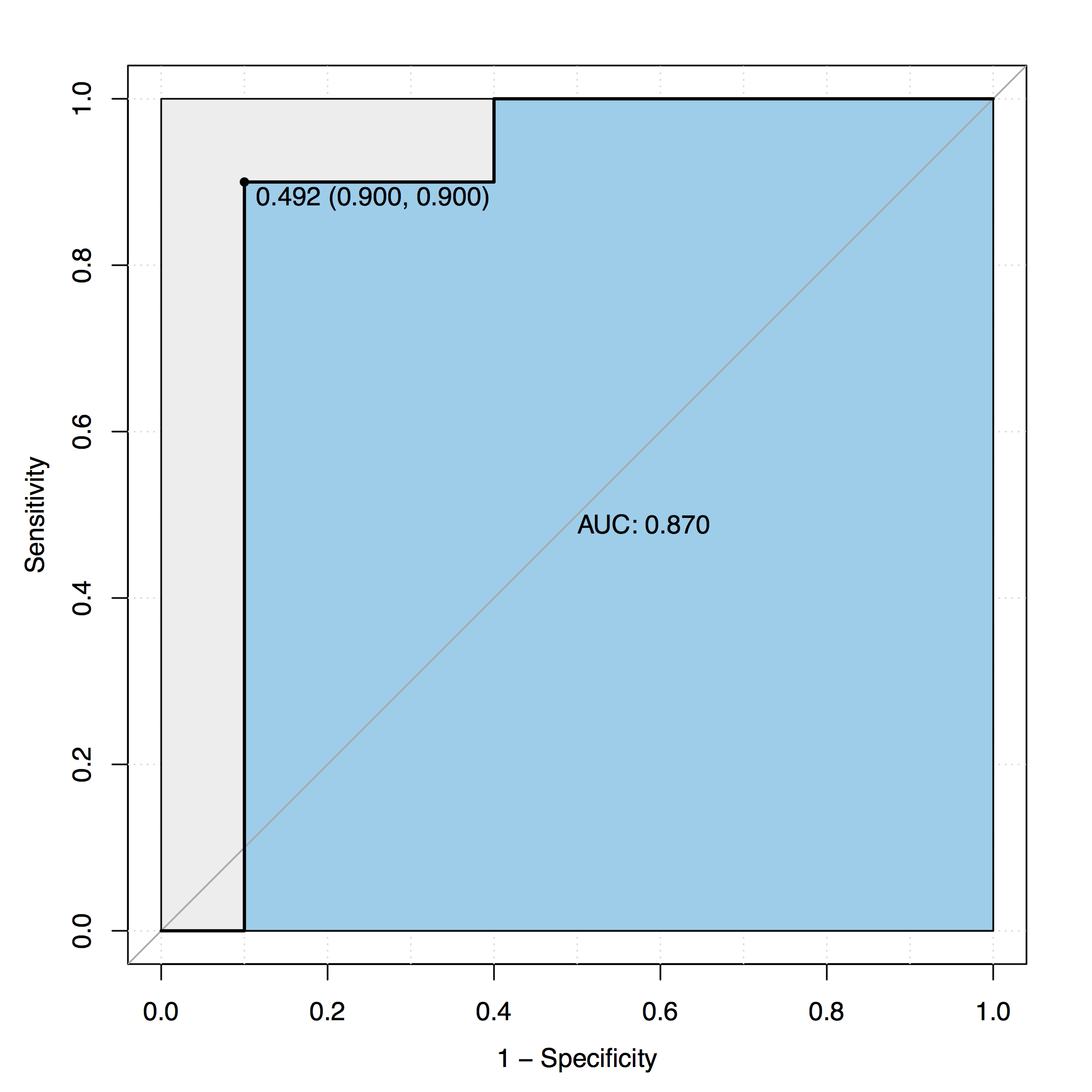

Supplement: FIGURE S7 — Based on two key bacterial taxa (V45, V124), the random forest prediction obtained an accurate rate with the ROC curve at AUC = 0.87. [file Image_7.JPEG]

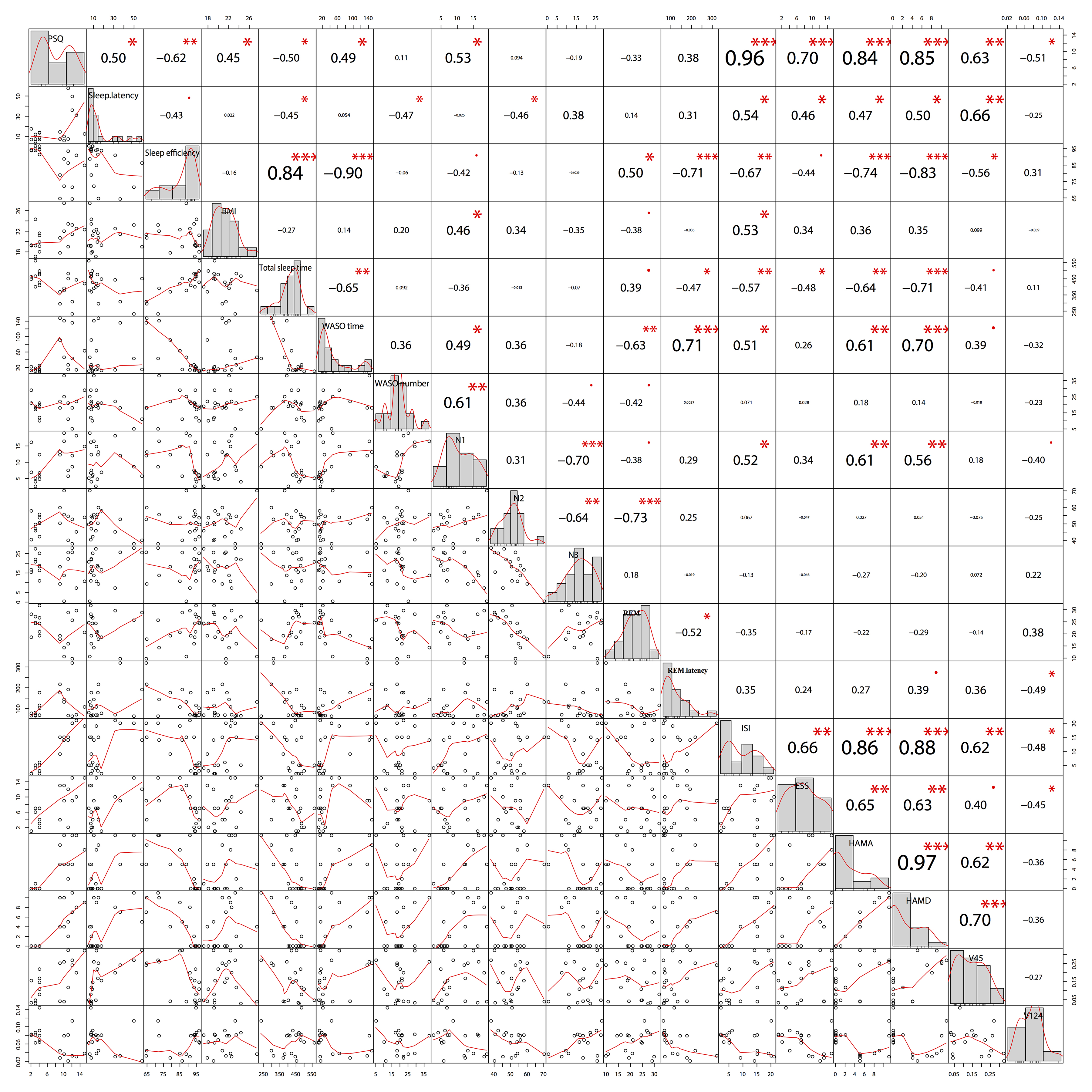

Supplement: FIGURE S8 — The detailed results of correlation analysis, these key taxa and clinical sleep parameter were mapped. [file Image_8.JPEG]

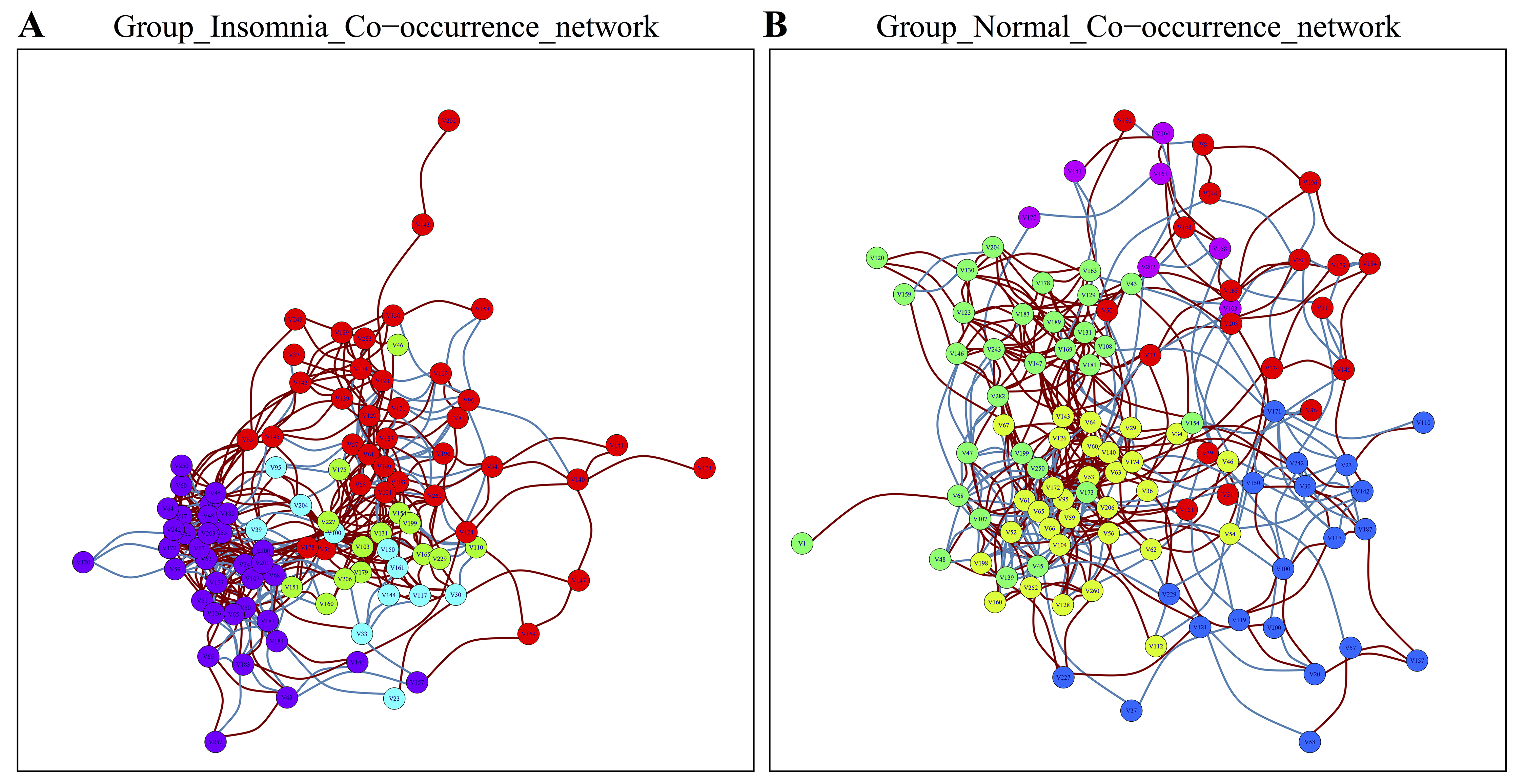

Supplement: FIGURE S9 — Co-occurrence network in each group plotted with “auto layout” parameter in “igraph” packages shows V45 and V124 occupied hub-like position in each network. [file Image_9.JPEG]

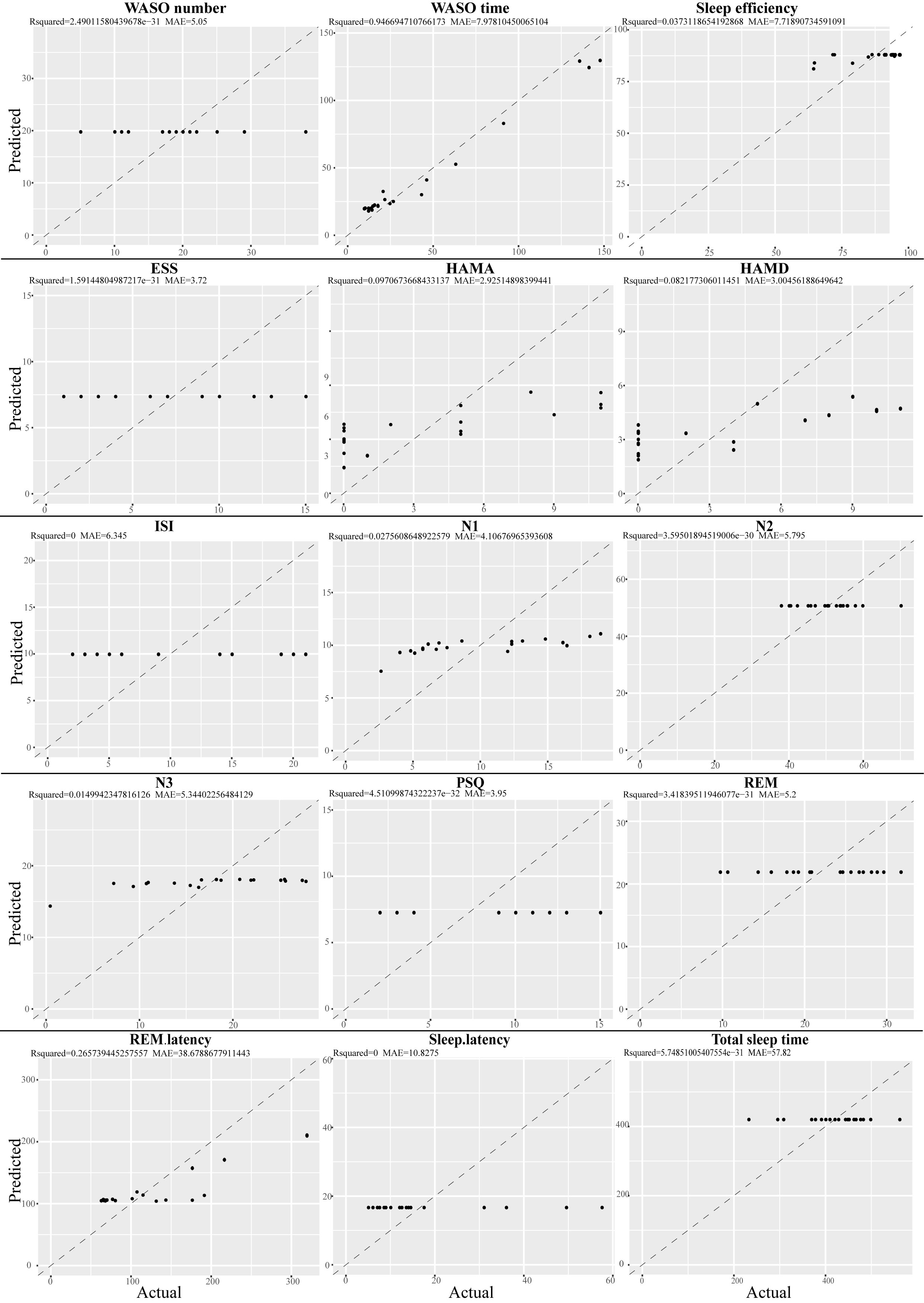

Supplement: FIGURE S10 — LASSO regression model to utilize the relative abundance of bacterial taxa to predict clinical sleep parameter. [file Image_10.JPEG]
