# Supplementary material for: Gut Microbiota as an Objective Measurement for Auxiliary Diagnosis of Insomnia Disorder
Source: Front Microbiol. 2019 Aug 13;10:1770. doi: 10.3389/fmicb.2019.01770 (PMC6701205; doi:10.3389/fmicb.2019.01770)
Supplement: Supplementary file 11 [file Table_1.DOCX]

**Inclusion and exclusion criteria of participants**

**Include criterion**

For insomnia group

1. The subject whose age was between 18 and 60 years old;
2. The subject who had accepted Diagnostic Interview for Sleep Patterns and

Disorder (DISP) Interview and met the diagnostic criteria for insomnia disorder in International classification of sleep disorders (ICSD, third edition);

1. The subject who had excluded other mental illnesses such as bipolar disorder and anxiety disorders, with Mini-International Neuropsychiatric Interview;
2. The subject whose insomnia symptoms had lasted more than 3 months.

For normal group

1. The subject whose age was between 18 and 60 years old;
2. The subject who had accepted DISP and excluded sleep disorder according to ICSD-3;
3. The subject who had excluded other mental illnesses such as bipolar disorder and anxiety disorders, with Mini-International Neuropsychiatric Interview;
4. The subject who had not any organic diseases which may affect experiment.

**Exclude criterion**

1. The subject whose age was not between 18 and 60 years old;
2. The subject who was a woman during menstruation, pregnant or lactation in experiment;
3. The subject who had diagnosed with other mental illnesses;
4. The subject who had any drug intake record in 3 months;
5. The subject who had habit of supplementing probiotics;
6. The subject who had received antibiotic treatment in six months;
7. The subject who experienced serious diarrhea or astriction in six months.

**Volunteer enrollment**

The experiment was proved by the Ethics Committee of Jinan University and recruited volunteers in public and The First Affiliated Hospital of Jinan University. After informed the rights and obligation, all participants understood benefit and risk in experiment totally and signed informed consent document. Comply with strict standards for inclusion and exclusion criteria, all participants were assessed by two psychiatrists, respectively. In the event of any dispute or difference of judgement, the participant would be excluded.

**Human fecal sample collection and DNA extraction**

All participants provided fresh stool samples at 7:00-8:30 am after polysomnography treatment, which were collected with a sterilized 1.5ml Eppendorf tube and frozen in -80°C freezer immediately. Bacterial DNA from patients’ feces was extracted by utilizing the ZR Fecal DNA Kit (Zymo Research, USA). A multiplexed amplicon library covering the V3-V4 region of 16S rDNA gene was PCR-amplified with the optimized primer sets for the Illumina HiSeq 2500 sequencing instrument (Primer:5'-ACTCCTACGGGAGGCAGCA-3'; 5'- GGACTACHVGGGTWTCTAAT-3').
